# Hnf1aos1 as a Metabolic Coordinator of Hepatic Lipid Homeostasis and Feedback Control

**DOI:** 10.3390/ncrna12030015

**Published:** 2026-04-30

**Authors:** Beshoy Armanios, Jing Jin, Ankit P. Laddha, Le Tra Giang Nguyen, Sherouk M. Tawfik, Neha Mishra, Jose E. Manautou, Xiao-Bo Zhong

**Affiliations:** 1 Department of Pharmaceutical Sciences, School of Pharmacy and Pharmaceutical Sciences, University of Connecticut, Storrs, CT 06269, USA; beshoy.armanios@uconn.edu (B.A.); jingjin.w@uconn.edu (J.J.); apladdha@uconn.edu (A.P.L.); le_tra_giang.nguyen@uconn.edu (L.T.G.N.); sherouk.tawfik@uconn.edu (S.M.T.); jose.manautou@uconn.edu (J.E.M.); 2 Department of Pathobiology, University of Connecticut, Storrs, CT 06269, USA; neha.mishra@uconn.edu

**Keywords:** lipid metabolism, hepatic steatosis, lipid dysregulation, lncRNA Hnf1aos1, metabolic feedback

## Abstract

**Background:** Long noncoding RNAs (lncRNAs) have emerged as critical regulators of hepatic metabolism and disease progression. The hepatocyte nuclear factor 1 alpha antisense 1 (HNF1A-AS1) lncRNA modulates liver-specific transcription factors; however, its physiological role in diet-dependent lipid homeostasis remains poorly defined. **Methods:** In this study, we investigated the mouse ortholog, Hnf1a opposite strand 1 (Hnf1aos1), using AAV-mediated knockdown in C57BL/6J mice fed either a chow diet (10% kcal from fat) or a high-fat diet (HFD; 60% kcal from fat) for 12 weeks. Metabolic phenotyping included hepatic lipid quantification, histological analysis, serum biochemistry, and quantitative gene expression profiling. **Results:** Loss of Hnf1aos1 produced distinct, diet-dependent alterations in hepatic lipid handling. Under chow conditions, knockdown mice exhibited selective hepatic cholesterol accumulation (6.10 ± 2.9 mg/g tissue vs. 3.51 ± 1.1 mg/g in controls), accompanied by dysregulation of cholesterol clearance pathways. In contrast, under HFD conditions, knockdown precipitated severe macrovesicular degeneration, with hepatic triglyceride levels approximately doubled relative to HFD-fed controls (51.72 ± 19.8 mg/g vs. 26.34 ± 11.9 mg/g) and a numerically elevated triglyceride-to-cholesterol ratio (TG:TC ≈ 6.1:1; *p* = 0.0621, trend). Chow/Kd mice gained significantly less weight than chow-fed controls, whereas HFD/Kd mice exhibited weight gain comparable to HFD controls despite severe hepatic steatosis. This paradoxical phenotype suggests impaired metabolic feedback at the post-transcriptional level, in which compensatory upregulation of *Hnf1a* mRNA is insufficient to suppress lipid-associated genes such as Cd36, despite profound lipid overload; however, HNF1A protein levels were not directly measured in this study. **Conclusion:** Collectively, these findings identify Hnf1aos1 as a regulator of hepatic lipid homeostasis whose loss produces a phenotype consistent with inappropriate lipid accumulation during nutrient excess, without defining the underlying molecular mechanism. Our results support a role for Hnf1aos1 in shaping hepatic metabolic plasticity and provide insight into lncRNA-associated MASLD phenotypes.

## 1. Introduction

Metabolic dysfunction-associated steatotic liver disease (MASLD) is the most common cause of chronic liver disease worldwide, affecting 25–38% of the global population, with some projections indicating that U.S. adult prevalence may exceed 55% by 2040 [1,2,3,4]. Despite hepatic triglyceride accumulation being the defining pathological feature, only 20–30% of individuals with steatosis progress to advanced fibrosis or cirrhosis [1,5,6]. This incomplete penetrance suggests that tissue-intrinsic regulatory mechanisms determine susceptibility to progressive disease.

A fundamental unresolved question is how hepatocytes sense lipid saturation and suppress lipid uptake and synthesis pathways during nutrient excess, a process termed metabolic feedback control. This sensing function in the liver requires integration of multiple nutrient-responsive pathways, including adenosine monophosphate-activated protein kinase (AMPK) signaling, which phosphorylates and inactivates acetyl-CoA carboxylase (ACC) to suppress de novo lipogenesis [7,8,9]; mTOR signaling, which coordinates sterol regulatory element-binding protein (SREBP) activation with nutrient availability [10,11,12]; and transcription factor-mediated sterol- and fatty acid-sensing mechanisms involving SREBP, ChREBP, and liver X receptor alpha (LXR) [13,14]. When these mechanisms fail, hepatocytes lose the ability to inhibit lipid uptake, creating conditions favorable for severe steatosis. While these nutrient-sensing pathways have been extensively characterized, emerging evidence suggests that long noncoding RNAs (lncRNAs) function as critical modulators that coordinate transcriptional responses within these networks [15,16], yet their physiological roles in diet-dependent metabolic feedback remain largely unexplored.

LncRNAs function as sophisticated regulators of hepatocyte metabolism. Some function as scaffolds for chromatin-modifying complexes [17,18], while others sequester microRNAs [19,20], and some modulate chromatin topology at metabolic gene loci [21,22]. However, the in vivo functional roles of most metabolic lncRNAs, particularly those linked to human genetic risk, remain uncharacterized in physiologically relevant diet-response models.

Hepatocyte nuclear factor 1 alpha (HNF1A) opposite strand 1 (Hnf1aos1), the murine ortholog of human HNF1A antisense 1 (HNF1A-AS1), is transcribed antisense to the hepatocyte-enriched transcription factor HNF1A. Both lncRNAs are evolutionarily conserved and participate in *cis* and *trans* regulation of the *HNF1A* locus through chromatin modulation, promoter insulation, and protein stabilization [23,24,25]. Recent mechanistic studies demonstrate that HNF1A-AS1 RNA directly binds the HNF1A protein, blocking tripartite motif containing 25 (TRIM25)-mediated ubiquitination and proteasomal degradation and stabilizing HNF1A at the protein level [25]. Additionally, the HASTER promoter (the promoter of HNF1A antisense transcripts) functions as a *cis*-acting transcriptional stabilizer that modulates HNF1A promoter–enhancer interactions to maintain physiological HNF1A concentrations [23]. HNF1A functions as a critical metabolic coordinator, suppressing fatty acid uptake genes [26,27]. Rare HNF1A mutations cause maturity-onset diabetes of the young type 3 (MODY3), while genome-wide association studies (GWASs) link *HNF1A* polymorphisms to multiple metabolic traits [28,29]. Crucially, HNF1A-AS1 variants appear as risk loci in GWASs for dyslipidemia and MASLD progression, but the biological basis of these associations remains incompletely understood [28,30].

Here, we employed AAV-mediated Hnf1aos1 knockdown in chow- and high-fat diet (HFD)-fed mice to investigate how Hnf1aos1 loss shapes diet-dependent hepatic lipid homeostasis. Our findings demonstrate that Hnf1aos1 knockdown produces distinct, diet-specific hepatic lipid phenotypes and systemic metabolic changes, revealing a paradoxical combination of severe hepatic steatosis under HFD conditions.

## 2. Results

### 2.1. Experimental Design and Successful Hnf1aos1 Knockdown In Vivo

**Figure 1:**
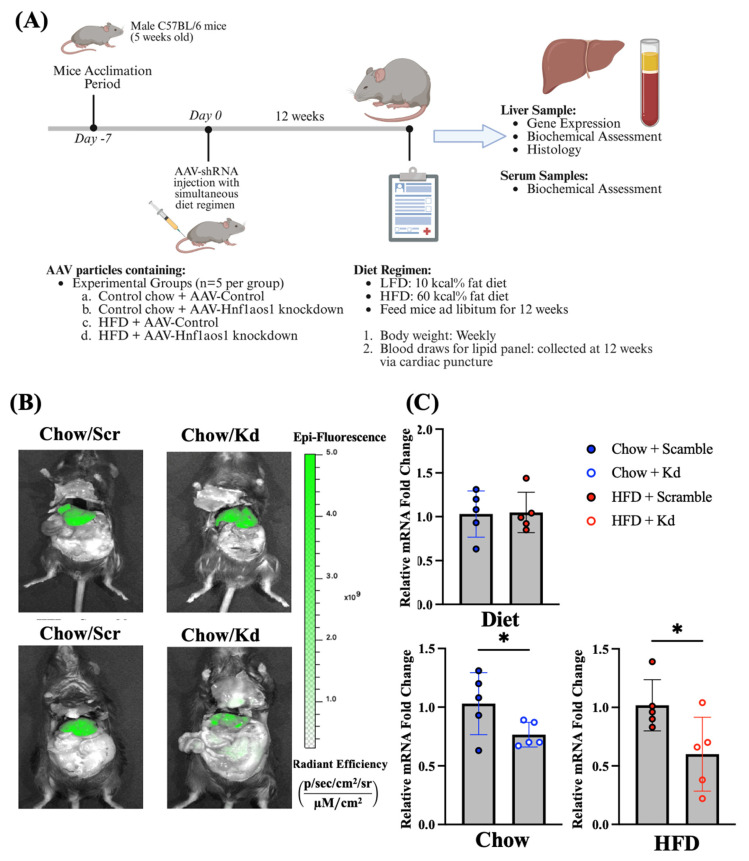
Experimental design and validation of hepatic Hnf1aos1 knockdown (Kd). (**A**) Schematic of the in vivo study design. Male C57BL/6J mice (5 weeks old) received tail vein injection of rAAV8-sh-Hnf1aos1 or rAAV8-sh-scramble at day 0 and were immediately randomized to a chow diet (10% kcal from fat) or a high-fat diet (HFD; 60% kcal from fat) for 12 weeks. Endpoint analyses were performed at week 12. (**B**) In vivo imaging system confirming hepatic AAV transduction at 12 weeks. Representative images are shown for all four experimental groups (Chow/Scr, Chow/Kd, HFD/Scr, and HFD/Kd). Total hepatic photon flux (photons/s) was quantified from a liver region of interest. (**C**) RT-qPCR validation of Hnf1aos1 knockdown. Top panel: Hnf1aos1 RNA expression in control mice (Chow/Scr and HFD/Scr). Bottom left: chow-fed mice (Chow/Scr vs. Chow/Kd). Bottom right: HFD-fed mice (HFD/Scr vs. HFD/Kd). Data are presented as fold change relative to diet-matched scrambled controls (mean ± SD, *n* = 5 per group; one-way ANOVA with Fisher’s LSD post hoc, * *p* < 0.05).

Male C57BL/6J mice (5 weeks old) were transduced with rAAV8 vectors targeting Hnf1aos1 (rAAV8-sh-Hnf1aos1) or the scrambled control (rAAV8-sh-scramble) and immediately assigned to either a chow diet (10% kcal fat) or an HFD (60% kcal fat) for 12 weeks (Figure 1A). Hepatic AAV transduction was confirmed by in vivo fluorescence imaging, demonstrating robust and comparable hepatic localization across all experimental groups, independent of dietary conditions (Figure 1B). Quantitative RT-qPCR validation confirmed that Hnf1aos1 expression was stable across dietary conditions in control mice with no significant difference between the chow and HFD/Scr groups (Figure 1C, top panel). Significant knockdown was achieved in both dietary conditions, with Chow/Kd mice showing an approximately 30% reduction and HFD/Kd mice showing an approximately 40% reduction in Hnf1aos1 RNA compared to their respective diet-matched controls (Figure 1C, bottom panels).

### 2.2. Diet-Dependent Hepatic Lipid Accumulation in Hnf1aos1 Knockdown Mice

**Figure 2:**
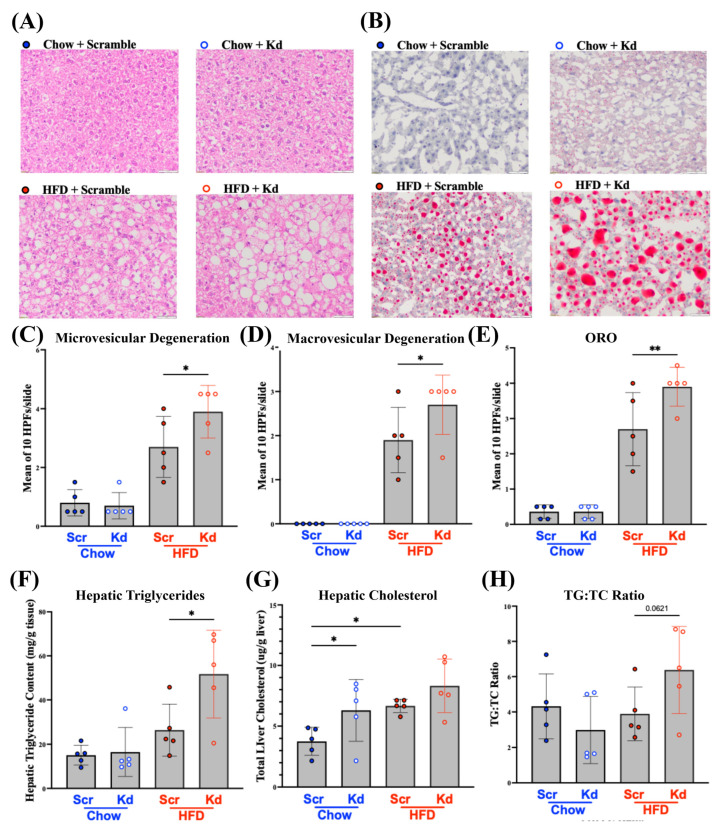
Hepatic lipid accumulation following Hnf1aos1 knockdown (Kd). (**A**) Representative H&E staining of mouse liver sections from Chow/Scr, Chow/Kd, HFD/Scr, and HFD/Kd mice. (**B**) Representative Oil Red O staining of mouse liver sections. Scale bar = 100 µm. (**C**) Microvesicular degeneration quantified from 10 HPFs per slide from H&E staining. (**D**) Macrovesicular degeneration quantified from 10 HPFs per slide from H&E staining. (**E**) Oil Red O-positive lipid area and intensity quantified from 10 HPFs per slide. (**F**) Hepatic triglycerides. (**G**) Hepatic cholesterol. (**H**) Triglyceride-to-cholesterol ratio (TG:TC). Data are presented as the mean ± SD (*n* = 5 per group; one-way ANOVA followed by Fisher’s LSD; * *p* < 0.05, ** *p* < 0.01). X-axis labels in panels (**C**–**H**) denote experimental groups: Chow/Scr, Chow/Kd, HFD/Scr, and HFD/Kd.

Histological analysis revealed clear diet- and genotype-dependent differences in hepatic lipid accumulation (Figure 2). In Chow/Scr mice, hematoxylin and eosin (H&E) and Oil Red O staining showed largely preserved hepatic architecture with minimal lipid deposition (Figure 2A,B). Microvesicular steatosis, defined by hepatocytes containing numerous tiny lipid droplets with centrally located nuclei, was modestly increased in chow-fed Hnf1aos1 knockdown livers, which displayed discrete microvesicular degeneration with small but discernible lipid droplets (Figure 2C). Under HFD conditions, scrambled mice developed the expected microvesicular steatosis and increased the Oil Red O-positive area relative to Chow/Scr controls. In contrast, HFD-fed Hnf1aos1 knockdown mice exhibited the most severe phenotype, with extensive mixed micro- and macrovesicular steatosis; macrovesicular change was characterized by single large lipid droplets that displaced the nucleus to the cell periphery, often forming dense confluent lipid aggregates (Figure 2B). Quantitative analysis confirmed a significant increase in macrovesicular lipid droplets in HFD/Kd livers compared to HFD controls (Figure 2D), indicating more severe hepatocellular lipid dysregulation.

Across groups, the pattern and severity of hepatocellular vacuolization on H&E staining closely paralleled neutral lipid accumulation on Oil Red O staining, with the lowest signal in Chow/Scr livers, minimal lipid deposition in Chow/Kd livers, an expected increase in HFD-scrambled livers, and the highest burden in HFD–knockdown livers.

Quantification of the Oil Red O-positive area and intensity confirmed these qualitative observations (Figure 2E). HFD feeding increased the hepatic lipid area in both scramble and knockdown mice compared to their respective chow-fed groups. Under HFD conditions, Hnf1aos1 knockdown mice accumulated significantly greater Oil Red O-positive area and intensity than HFD/Scr controls, indicating increased lipid accumulation specifically when Hnf1aos1 was suppressed under excess nutrient conditions. A semi-quantitative histopathology scoring table is provided below (Table 1).

Biochemical analysis of hepatic lipid composition revealed two distinct patterns across dietary conditions. Under chow conditions, Hnf1aos1 loss resulted in a selective elevation of hepatic cholesterol, while hepatic triglyceride content remained unchanged. Hepatic triglycerides did not differ between chow-fed knockdown and scramble controls (15.05 ± 4.5 vs. 16.45 ± 11.0 mg/g) (Figure 2F), whereas hepatic cholesterol was significantly higher in knockdown mice (6.10 ± 2.9 mg/g) than in chow-fed scramble controls (3.51 ± 1.1 mg/g) (Figure 2G).

Under the HFD challenge, this profile shifted dramatically. Hepatic triglycerides in knockdown mice reached 51.72 ± 19.8 mg/g, which is approximately two-fold higher than in HFD/Scr controls (26.34 ± 11.9 mg/g) (Figure 2F), and hepatic cholesterol was further elevated to 8.47 ± 2.5 mg/g compared to 6.67 ± 0.6 mg/g in HFD-fed controls (Figure 2G). The selective increase of triglycerides in knockdown livers produced an elevated triglyceride-to-cholesterol ratio (TG:TC = 6.1:1) compared with HFD/Scr mice (TG:TC = 4.0:1) (Figure 2H); however, this difference did not reach statistical significance (one-way ANOVA, *p* = 0.0621) and should be interpreted as a trend. While the direction and magnitude of the difference are consistent with disproportionate triglyceride accumulation, formal statistical significance was not achieved, likely due to the high within-group variance in the HFD/Kd group (51.72 ± 19.8 mg/g). Mean ± SD TG:TC values for all groups are provided below (Table 2).

These biochemical and histological findings demonstrate that Hnf1aos1 knockdown produces diet-dependent hepatic lipid accumulation, with a shift from selective cholesterol elevation on chow to triglyceride-dominant steatosis on HFDs. To determine the transcriptional mechanisms underlying these phenotypes, we next profiled the hepatic expression of genes governing lipid uptake and synthesis, cholesterol clearance, and lipoprotein metabolism.

### 2.3. Dysregulation of Master Regulators, Lipid Uptake, and Lipid Synthesis

**Figure 3:**
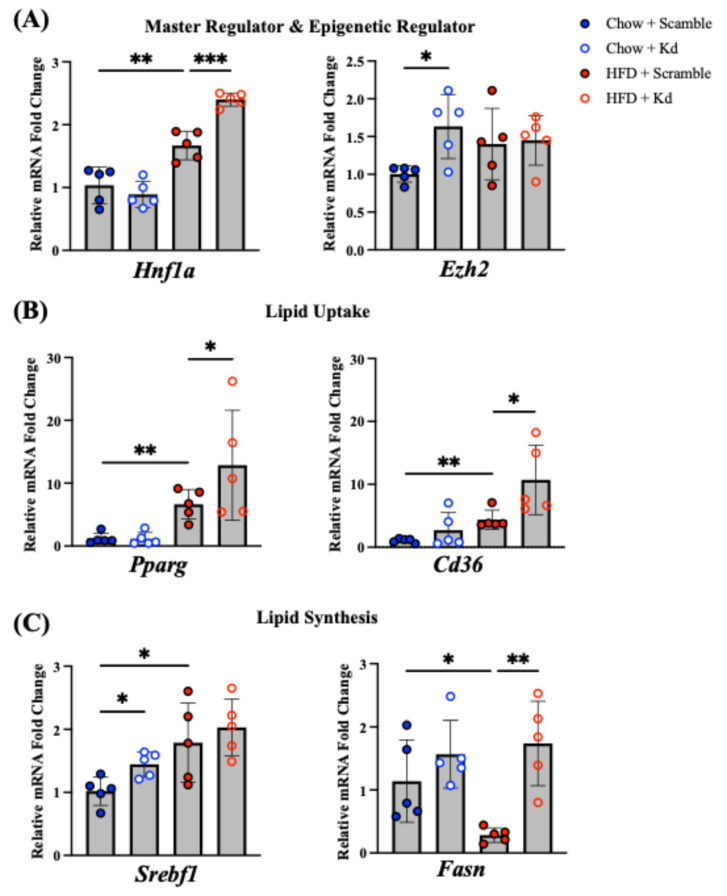
Hepatic gene expression of master regulators, lipid uptake, and lipid synthesis. (**A**) mRNA expression of *Hnf1a* (**left**) and *Ezh2* (**right**). (**B**) mRNA expression of *Pparg* (**left**) and *Cd36* (**right**). (**C**) mRNA expression of *Srebf1* (**left**) and *Fasn* (**right**). All mRNAs were measured by RT-qPCR, normalized to *Ppia*, and expressed as fold change relative to Chow/Scr controls. Data are presented as the mean ± SD (*n* = 5 per group; one-way ANOVA followed by Fisher’s LSD; * *p* < 0.05, ** *p* < 0.01, and *** *p* < 0.001).

**Figure 4:**
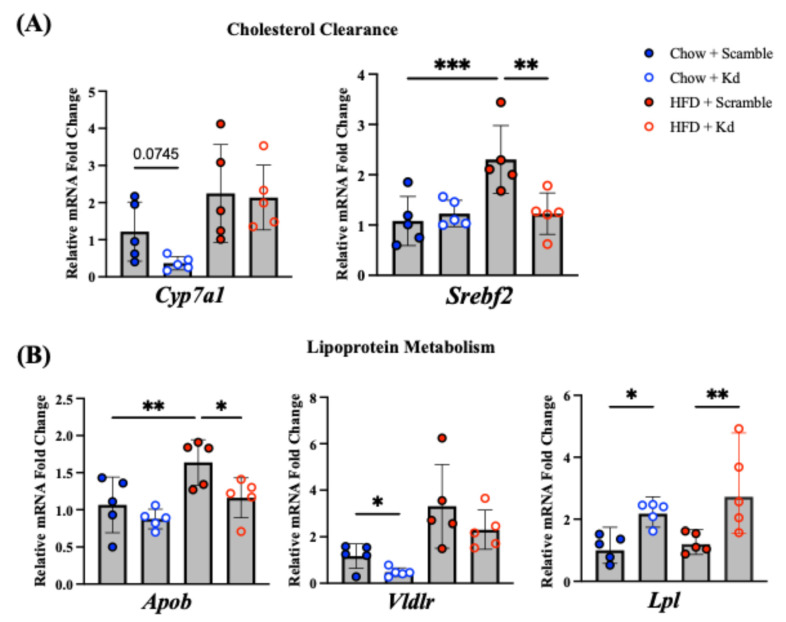
Hepatic gene expression in cholesterol clearance and lipoprotein metabolism. (**A**) Cholesterol Clearance: mRNA expression of *Cyp7a1* and *Srebf2*. (**B**) Lipoprotein Metabolism: mRNA expression of *Apob*, *Lpl*, and *Vldlr*. All mRNAs were measured by RT-qPCR, normalized to *Ppia*, and expressed as fold change relative to Chow/Scr controls. Data are presented as the mean ± SD (*n* = 5 per group; one-way ANOVA followed by Fisher’s LSD; * *p* < 0.05, ** *p* < 0.01,*** *p* < 0.001).

To identify the transcriptional basis of the diet-dependent lipid phenotypes associated with Hnf1aos1 deficiency described above, we profiled key lipid metabolism genes by RT-qPCR. Genes governing lipid uptake, de novo lipogenesis, and epigenetic regulation are presented in Figure 3, while cholesterol clearance and lipoprotein metabolism genes are presented in Figure 4.

In Figure 3A, the transcriptional regulator *Hnf1a* showed the highest expression among all four experimental groups in HFD/Kd mice, with *Hnf1a* mRNA significantly elevated compared with HFD-fed controls. In contrast, the epigenetic regulator enhancer of Zeste 2 (*Ezh2*, a histone methyltransferase component of PRC2) was significantly upregulated only in Chow/Kd mice and returned to basal levels under HFD conditions.

In the lipid uptake pathway (Figure 3B), *Cd36* and *Pparg* showed no significant changes under chow conditions. However, under the HFD challenge, both *Cd36* and *Pparg* were significantly upregulated in knockdown livers compared with HFD-fed controls, consistent with increased fatty acid uptake signaling during nutrient excess. In keeping with this pattern, *Fabp4* (fatty acid binding protein 4) was significantly increased in HFD-fed groups compared with chow groups (Supplementary Figure S1).

In the lipid synthesis pathway (Figure 3C), *Srebf1* was significantly upregulated in Chow/Kd mice. Under HFD conditions, *Fasn* expression, which was reduced in HFD-fed controls, was significantly de-repressed (restored) in HFD/Kd mice, indicating failure to maintain lipogenic suppression during nutrient excess. Extended profiling showed that *Acaca* (acetyl-CoA carboxylase alpha) exhibited a similar upregulation trend in HFD-fed groups, while *Lxra* (liver X receptor alpha) expression was significantly elevated by HFD feeding, with HFD/Kd mice maintaining levels comparable to those of HFD-fed controls (Supplementary Figure S1). Additionally, *Ppara* (peroxisome proliferator-activated receptor alpha) was significantly upregulated in both HFD groups compared with chow controls (Supplementary Figure S1).

### 2.4. Dysregulation of Cholesterol and Lipoprotein Metabolism

Having established that Hnf1aos1 knockdown dysregulates lipid uptake and synthesis pathways (Figure 3), we next examined whether cholesterol clearance and lipoprotein metabolism were similarly affected (Figure 4). Analysis of cholesterol clearance genes revealed distinct patterns. *Cyp7a1*, which catalyzes the rate-limiting step in bile acid synthesis, was reduced in Chow/Kd mice with a strong trend toward statistical significance (*p* = 0.0745) (Figure 4A). In the sterol regulatory axis, HFD treatment significantly induced *Srebf2* expression in control mice compared with chow-fed controls, whereas HFD/Kd mice showed significant suppression of *Srebf2* expression compared with HFD-fed controls (Figure 4A).

In the lipoprotein assembly and processing pathway, *Apob* was significantly suppressed in HFD/Kd mice, while *Lpl* (lipoprotein lipase) levels were significantly elevated in both chow and HFD/Kd groups (Figure 4B). Finally, analysis of lipoprotein receptors revealed opposing responses: *Vldlr* (very-low-density lipoprotein receptor) was significantly downregulated in Chow/Kd mice (Figure 4B), whereas *Ldlr* (LDL receptor) expression was significantly upregulated in HFD-fed groups compared with chow controls, with HFD/Kd mice maintaining this approximately 2-fold increase in transcript levels (Supplementary Figure S1). *Abca1* (ATP-binding cassette transporter A1) expression showed no significant differences across dietary or genotypic groups (Supplementary Figure S1).

### 2.5. Systemic Metabolic Parameters

**Figure 5:**
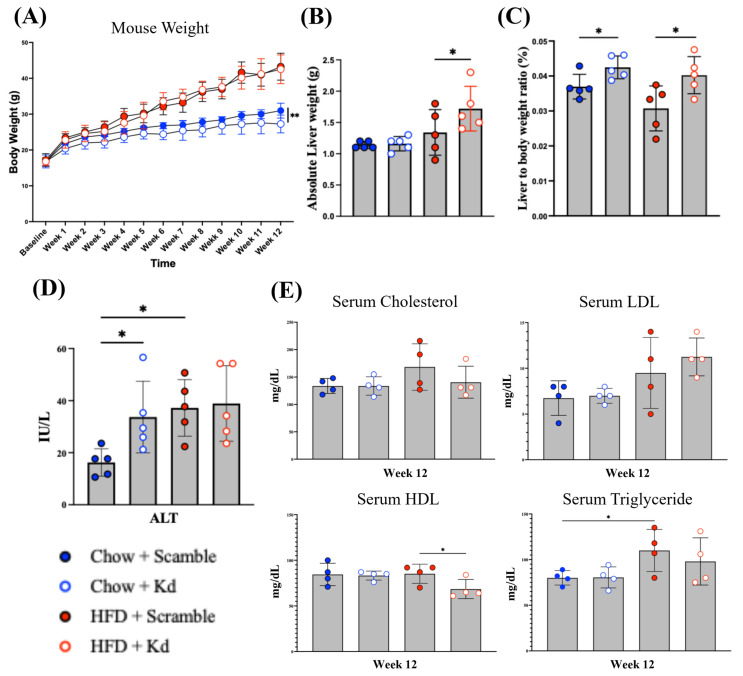
Systemic metabolic characterization. (**A**) Body weight trajectories over 12 weeks of dietary intervention with body weight measured weekly in all four experimental groups. Data are means ± SDs (*n* = 5 per group) and were analyzed using a linear mixed-effects model (REML; GraphPad Prism 9.0) with time as a within-subject factor, revealing significant main effects of time (*p* < 0.0001) and group (*p* < 0.0001), as well as a significant time × group interaction (*p* < 0.0001); pairwise differences were evaluated by Fisher’s LSD post hoc test. (**B**) Absolute liver weight at study termination (week 12 of diet, week 14 post-AAV injection). (**C**) Liver-to-body-weight ratio (%) at study termination. (**D**) Serum alanine aminotransferase (ALT, U/L) at study termination, measured by the colorimetric assay. Data are presented with Fisher’s LSD post hoc test confirming pairwise differences and shown as means ± SDs (*n* = 5 per group; one-way ANOVA, * *p* < 0.05). (**E**) Serum lipid panel at study termination, reported as means ± SDs, with n = 4 per group (one sample per group was excluded due to insufficient serum volume obtained during cardiac puncture). One-way ANOVA is used to determine significant differences; * *p* < 0.05, ** *p* < 0.01.

The hepatic transcriptional and lipid composition data (Figure 2, Figure 3 and Figure 4) established that Hnf1aos1 knockdown produces severe intrahepatic lipid dysregulation. To determine whether this hepatic phenotype manifested systemically, we evaluated body weight, liver weight, serum transaminases, and circulating lipid profiles (Figure 5).

Mixed-effects analysis of longitudinal body weight, with mouse treated as a random effect and time, group, and their interaction treated as fixed effects, revealed significant main effects of time (*p* < 0.0001) and group (*p* < 0.0001), as well as a significant time × group interaction (*p* < 0.0001), indicating diet- and genotype-specific divergence in weight gain patterns over the 12-week study (Figure 5A). Although HFD feeding drove the expected weight gain in all groups, Chow/Kd mice gained significantly less weight than their scrambled counterparts. At week 12, terminal body weights were 30.98 ± 2.07 g for Chow/Scr and 27.26 ± 2.49 g for Chow/Kd mice, confirming reduced weight gain in Chow/Kd animals (** *p* < 0.01). In contrast, HFD/Kd mice gained weight at a rate indistinguishable from that of the HFD controls, with comparable terminal body weights (43.24 ± 3.75 g vs. 42.52 ± 4.01 g).

Examination of the gross liver morphology revealed significant hepatomegaly. Absolute liver weight was markedly increased by HFD feeding, with both HFD groups exhibiting significantly greater liver weights compared to chow controls (Figure 5B). This organ enlargement was confirmed by the liver-to-body weight ratio (Figure 5C), which was elevated in the HFD-fed groups, consistent with the severe macrovesicular steatosis observed histologically.

Serum biochemistry results were compatible with mild hepatocellular stress and functional dysregulation. ALT levels were significantly affected by both diet and genotype (Figure 5D), although all group means remained within a range (≤40 IU/L) not typically associated with overt hepatocellular injury. Chow/Kd, HFD/Scr, and HFD/Kd mice all showed similarly elevated ALT levels relative to chow-fed scramble controls, indicating that ALT was upregulated in the knockdown and/or HFD groups compared with baseline chow + Scr.

Comprehensive serum lipid analysis (Figure 5E) revealed selective dysregulation of HDL metabolism with otherwise preserved systemic lipid profiles. Serum cholesterol and LDL cholesterol levels showed a trend toward higher levels in HFD-fed groups than in chow-fed groups, but these differences did not reach statistical significance, and there was no significant difference between HFD/Kd and HFD/Scr mice (top panels). In contrast, serum HDL cholesterol was significantly reduced in HFD/Kd mice compared with HFD/Scr controls (* *p* < 0.05, Fisher’s LSD), with HDL levels in HFD/Kd mice approximating those observed in chow-fed groups (bottom-left panel). Serum triglycerides were the only lipid parameter significantly increased by diet, with HFD-fed mice exhibiting higher triglyceride levels than chow-fed mice, irrespective of genotype (bottom-right panel). The lack of a genotype effect on serum triglycerides despite massive hepatic triglyceride accumulation is consistent with *Apob* suppression and impaired VLDL secretion, which trap triglycerides within hepatocytes rather than exporting them to the circulation.

## 3. Discussion

Our study demonstrates that under chow conditions, Hnf1aos1 knockdown resulted in selective elevation of hepatic cholesterol, accompanied by reduced *Cyp7a1* expression and significant *Vldlr* downregulation, while hepatic triglyceride content and lipid uptake gene expression (*Cd36*, *Pparg*) remained unchanged. Under the HFD challenge, Hnf1aos1 knockdown produced marked hepatic triglyceride accumulation with further cholesterol elevation, resulting in an increased TG:TC ratio relative to HFD controls. These changes were accompanied by significant upregulation of *Cd36* and *Pparg* compared to HFD-fed controls, restoration of *Fasn* expression despite HFD feeding, maximal Hnf1a transcription, suppressed *Srebf2* expression, elevated *Lpl* levels, and reduced *Apob* expression. Systemically, HFD/Kd mice exhibited the highest ALT levels among all groups and a selective reduction in serum HDL cholesterol compared to HFD controls.

### 3.1. Hnf1aos1 as a Metabolic Coordinator of Hepatic Lipid Homeostasis

This study establishes Hnf1aos1 as a novel lncRNA regulator of diet-responsive hepatic metabolic plasticity. The human ortholog HNF1A-AS1 stabilizes the HNF1A protein by directly binding the HNF1A DNA binding domain and blocking TRIM25-mediated ubiquitination and proteasomal degradation. Our transcriptional data are consistent with the hypothesis that Hnf1aos1 may operate through analogous mechanisms in murine hepatocytes; however, HNF1A protein levels were not directly assessed in the present study, and this mechanistic parallel remains to be confirmed [25,31].

It is worth noting that hepatic Hnf1aos1 knockdown was partial in this study (~30% under chow conditions and ~40% under HFD conditions). Despite this incomplete silencing, we observed robust and biologically coherent phenotypic outcomes across histological, biochemical, and transcriptional endpoints, consistent with prior work showing that partial knockdown is sufficient to drive transcriptional, proteomic, and histological changes [32]. The dose-dependent nature of lncRNA biology suggests that partial loss-of-function is sufficient to unmask phenotypes when the target lncRNA regulates rate-limiting steps in metabolic circuitry. Consistent with this interpretation, haploinsufficiency models for hepatic transcription factors such as HNF1A are well established in producing metabolic phenotypes in mice and humans. Accordingly, partial knockdown (~30–40%) may be sufficient to reduce HNF1A protein stabilization below the threshold required for effective feedback suppression during HFD-induced nutrient excess. Future studies using complete knockout models or higher-efficiency siRNA delivery will be important for establishing a dose–response relationship.

The observation that chow-fed knockdown mice gained significantly less weight than chow-fed controls, while HFD-fed knockdown mice gained weight indistinguishably from HFD controls, reveals a diet-dependent systemic metabolic effect of Hnf1aos1 loss. This pattern is consistent with HNF1A-deficient mouse models, in which *Hnf1a*–null mice exhibit Laron-type dwarfism with body weights approximately 50–60% lower than those of heterozygous littermates, attributable to reduced hepatic IGF-I expression and growth hormone resistance. The reduced weight gain in our chow-fed knockdown mice may reflect multiple converging mechanisms. Transcriptional changes suggestive of reduced de novo lipogenesis (lower *Srebf1* and *Fasn*), possible alterations in bile acid synthesis pathways (CYP7A1), and a catabolic shift consistent with partial HNF1A protein insufficiency may collectively contribute to this phenotype. Under HFD conditions, however, the dietary caloric surplus overrides these catabolic tendencies: dysregulated fatty acid uptake (elevated *Cd36* and *Pparg*) and hepatic lipid trapping (elevated *Lpl* and suppressed *Apob*) may promote lipid accumulation that maintains body weight at HFD-control levels despite hepatic metabolic dysfunction. This diet-dependent body weight phenotype supports the model that Hnf1aos1 loss impairs adaptive responses, with phenotypic consequences that depend on the nutritional context rather than reflecting a fixed metabolic defect [33].

Our data suggest a failure in metabolic feedback control. Hnf1aos1 loss disrupts HNF1A-dependent suppression of lipid uptake genes during nutrient excess. Under HFD conditions, Hnf1aos1 knockdown triggered paradoxical upregulation of *Cd36* and *Pparg*, along with restored *Fasn* expression, despite severe hepatic lipid overload and maximal *Hnf1a* mRNA transcription. This dissociation between transcriptional abundance and functional suppression indicates that Hnf1aos1 is required for functional coupling between Hnf1a mRNA abundance and HNF1A-dependent suppression of lipogenic and lipid uptake genes, consistent with a model in which Hnf1aos1 stabilizes the HNF1A protein, as demonstrated for the human ortholog HNF1A-AS1 [25]. Mechanistically, HNF1A functions as a transcriptional repressor of these uptake genes, and protein stabilization by Hnf1aos1, as shown for the human ortholog, is likely important for this repressive function during nutrient stress. However, direct HNF1A protein measurements in our model are still needed.

Under basal (chow) conditions, Hnf1aos1 loss produced selective cholesterol steady-state defects characterized by elevated hepatic cholesterol, reduced *Vldlr* expression (which mediates VLDL remnant uptake), and suppressed *Cyp7a1* expression (which catalyzes the rate-limiting step in bile acid synthesis) (*p* = 0.0745) [34,35,36]. Notably, fatty acid uptake genes (*Cd36*, *Pparg*) remained unchanged, suggesting distinct roles for Hnf1aos1 in cholesterol versus fatty acid homeostasis.

These findings position Hnf1aos1 as a critical cofactor for HNF1A-mediated metabolic control, demonstrating how lncRNAs function as essential regulators of transcription factor protein stability. This principle may explain why *HNF1A-AS1* variants are associated with MASLD risk in human populations [30,37].

### 3.2. Impaired Metabolic Feedback Control of Hepatic Lipid Uptake

Unlike MASLD models driven by excessive de novo lipogenesis, our data support a model in which metabolic feedback control of hepatic lipid uptake is impaired. The paradoxical upregulation of *Cd36* and *Pparg* mRNA in HFD/Kd mice, despite marked hepatic lipid overload and a distinct sterol-regulatory response (HFD-induced *Srebf2* in controls but Srebf2 suppression in knockdown mice), demonstrates that Hnf1aos1 loss selectively impairs fatty acid-sensing mechanisms. This dissociation between compensatory *Hnf1a* mRNA transcription and failure to suppress lipid uptake genes indicates that transcription factor abundance alone is insufficient for metabolic control.

These transcriptional findings are consistent with dysregulation of the HNF1A-PPARG axis, though direct mechanistic proof requires HNF1A protein quantification and chromatin occupancy assays. HNF1A functions as a direct transcriptional repressor of *PPARG*. It binds conserved HNF1A response elements (HREs) within the *PPARG* promoter to suppress expression [26]. Loss of HNF1A function leads to 10-fold induction of hepatic PPARG and subsequent upregulation of its target genes, including *CD36*, aP2, and lipoprotein lipase [26]. *CD36* is a well-established downstream target of PPARG, with peroxisome proliferator response elements (PPREs) in its promoter mediating direct transcriptional activation [38]. The coordinated upregulation of both *Pparg* and *Cd36* mRNA observed in HFD/Kd mice is therefore consistent with *Cd36* induction occurring downstream of PPARG of increased expression. Critically, genetic ablation of PPARG in HNF1A-deficient hepatocytes completely rescues hepatic steatosis, establishing PPARG as the obligate mediator of lipid dysregulation downstream of HNF1A loss [26]. This regulatory axis is further controlled by Akt2-mediated phosphorylation of HNF1A, which relieves PPARG suppression during nutrient excess [26].

Our findings are consistent with a working model in which Hnf1aos1 may stabilize HNF1A protein to maintain its repressive function on *Pparg* during nutrient saturation. In the absence of Hnf1aos1, HNF1A protein levels could potentially become insufficient to maintain repression of *Pparg* despite maximal mRNA transcription, leading to induction of *Pparg* and downstream lipid uptake genes even when hepatic lipid stores are saturated. We emphasize that this model is inferential. HNF1A protein levels were not measured in our study, and whether reduced HNF1A protein abundance is the proximate cause of *Pparg* de-repression in the murine system remains to be established by Western blot, immunofluorescence, or mass spectrometry–based protein quantification in future work. Definitive proof of HNF1A protein dysfunction requires chromatin immunoprecipitation, protein-level quantification, and functional rescue experiments.

Our data support a working model in which Hnf1aos1 modulates HNF1A-dependent control of the *Pparg*–*Cd36* lipid uptake axis and hepatic lipoprotein handling, thereby influencing whether hepatocytes appropriately downregulate triglyceride uptake during nutrient excess. This hypothesis is consistent with prior evidence that human HNF1A-AS1 stabilizes HNF1A protein but will require direct assessment of HNF1A protein levels, chromatin occupancy, and rescue experiments in the Hnf1aos1 knockdown model.

### 3.3. Hepatic Lipid Composition and Diet-Dependent Metabolic Defects

Hepatic lipid composition analysis revealed distinct phenotypic patterns reflecting diet-dependent dysregulation. Both control groups (Chow/Scr and HFD/Scr) maintained triglyceride-to-cholesterol (TG:TC) ratios of approximately 4.0:1, consistent with balanced lipid accumulation without metabolic dysregulation [39]. In contrast, HFD-fed Hnf1aos1 knockdown mice displayed a markedly elevated TG:TC ratio of 6.1:1, reflecting disproportionate triglyceride accumulation (51.72 ± 19.8 mg/g versus 26.34 ± 11.9 mg/g in HFD controls). This pattern of selective triglyceride expansion is consistent with MASLD driven by dysregulated hepatic lipid uptake rather than impaired triglyceride export or oxidation, though the TG:TC difference did not reach formal statistical significance (*p* = 0.0621) and this interpretation should be considered preliminary [40].

The numerically elevated hepatic TG:TC ratio (6.1 in HFD/Kd vs. 4.0 in HFD/Scr) suggests a trend toward triglyceride-dominant lipid accumulation, though this difference did not achieve statistical significance (*p* = 0.0621) at the present sample size, consistent with steatosis driven primarily by fatty acid uptake rather than de novo lipogenesis [40]. While specific TG:TC thresholds have not been established in preclinical models, this degree of disproportionate triglyceride expansion parallels clinical observations linking lipid composition shifts to impaired metabolic feedback [41].

The hepatic transcriptional signature aligns with this biochemical phenotype. The coordinated upregulation of *Cd36* and *Pparg* mRNA is consistent with the established PPARG to CD36 transcriptional axis, wherein CD36 is a direct PPARG target gene [42,43]. This coordinated expression suggests increased fatty acid uptake capacity, which is reflected biochemically in the elevated TG:TC ratio characteristic of steatosis dominated by fatty acid uptake. Notably, this transcriptional-biochemical correlation distinguishes uptake-driven MASLD from lipogenesis-dominant or export-deficient subtypes.

Clinically, these hepatic findings align with serum lipid biomarkers predictive of MASLD severity [39,44]. The serum TG/HDL-C ratio is a strong non-invasive marker of MASLD, with reported AUC values of approximately 0.73–0.85 for detecting steatosis across diverse cohorts. In a biopsy-based MASLD study, a TG/HDL-C cut-off value of 3.7 yielded an AUC of 0.747 for histologically confirmed disease, whereas other cohorts have identified lower optimal cut-offs (around 0.9–1.4) for ultrasound-defined MAFLD [45]. The disproportionate triglyceride accumulation and elevated TG:TC ratio in HFD/Scr livers suggest that this MASLD subtype may be identifiable by lipid ratio signatures in human patients, providing a potential biomarker for disease mechanism.

### 3.4. Diet-Specific Metabolic Defects

Hnf1aos1 loss produces distinct metabolic phenotypes under chow versus HFD conditions, revealing selective impairment of nutrient-dependent feedback control. Under chow conditions, altered cholesterol clearance, characterized by elevated hepatic cholesterol, suppressed *Cyp7a1* expression (*p* = 0.0745), and reduced *Vldlr*, occurs independently of HFD feeding. The coordinated dysregulation of both genes provides a transcriptional signature consistent with impaired cholesterol sensing and clearance. Critically, fatty acid uptake genes (*Cd36*, *Pparg*) remain unchanged under basal conditions, indicating intact lipid sensing for fatty acid metabolism. This dissociation suggests that Hnf1aos1 has distinct roles in cholesterol versus fatty acid homeostasis, with cholesterol clearance being selectively vulnerable to Hnf1aos1 loss even under nutrient-replete chow feeding.

Under HFD conditions, the lipid uptake and synthesis changes described above (Section 3.2) are accompanied by additional metabolic alterations that together define a diet-specific Hnf1aos1 knockdown phenotype. Compared with chow, the defect shifts from a predominantly cholesterol-selective disturbance to a state characterized by increased fatty acid uptake and triglyceride storage under nutrient excess. In this setting, *Ppara* expression is maintained or modestly increased, suggesting that transcriptional programs for fatty acid oxidation remain in place. Nevertheless, this adaptive response appears insufficient to prevent steatosis when hepatic fatty acid influx from uptake and synthesis chronically exceeds oxidative and export capacity; under such conditions, triglycerides can accumulate in the liver even in the presence of preserved or elevated *Ppara* expression. Consistent with this, PPARG has been shown to modulate lipid metabolism and steatosis in NAFLD and remains a major therapeutic target in this context [46].

### 3.5. Transcriptional-Functional Dissociation

Several observations indicate that Hnf1aos1 loss produces dissociation between transcriptional regulation and functional metabolic outcomes, suggesting that transcription factor function extends beyond direct transcriptional control. *Ldlr* upregulation did not prevent hepatic cholesterol accumulation, and unchanged *Abca1* mRNA did not maintain serum HDL cholesterol levels in HFD/Kd mice, indicating that mRNA abundance alone is insufficient to drive cholesterol homeostatic responses. LDLR and ABCA1 are extensively regulated post-transcriptionally through mechanisms independent of transcript levels: LDLR function is controlled by mRNA stability via RNA-binding proteins (ZFP36L1/L2), protein degradation via PCSK9-mediated lysosomal trafficking, and subcellular localization, while ABCA1 activity is regulated by protein–protein interactions governing ER-to-plasma membrane trafficking [47,48,49].

The dissociation between mRNA expression and metabolic function observed for *Ldlr* and *Abca1* parallels the broader pattern seen in this study, in which changes in transcript levels did not consistently predict lipid handling or steatosis severity. *Hnf1a* transcriptional upregulation in Hnf1aos1 knockdown livers was not sufficient to suppress lipid uptake genes or prevent triglyceride accumulation, suggesting that transcription factor mRNA abundance alone is an imperfect surrogate for metabolic control. These findings are consistent with human data implicating HNF1A-AS1 in the regulation of HNF1A protein, but the specific mechanisms linking Hnf1aos1 to HNF1A activity and downstream lipid regulators such as LDLR and ABCA1 were not directly tested in this study. Critically, HNF1A protein abundance was not measured; therefore, whether the transcriptional dissociation documented here reflects protein-level insufficiency or another post-transcriptional mechanism cannot be determined from the present data. Direct HNF1A protein quantification (Western blot or targeted proteomics) is a priority for future work.

### 3.6. Hepatic Lipid Trapping

The dysregulation of lipoprotein metabolism is consistent with an intrahepatic lipid trap that amplifies triglyceride accumulation. *Lpl* (lipoprotein lipase) upregulation concurrent with suppressed *Apob* creates opposing metabolic fluxes: LPL catalyzes circulating triglyceride hydrolysis and releases fatty acids for hepatic uptake, while ApoB is essential for VLDL assembly and triglyceride export [50,51]. This coordinated transcriptional pattern is consistent with a one-way metabolic valve favoring intrahepatic triglyceride accumulation, although direct assessment of VLDL secretion and hepatic lipid flux will be required to confirm this model.

Critically, this lipoprotein dysregulation occurs in the context of impaired metabolic feedback control. Under normal conditions, hepatic lipid saturation is expected to limit fatty acid influx, while maintaining triglyceride export capacity. In Hnf1aos1 knockdown mice, this coordinated suppression fails: *Lpl* remains elevated despite severe hepatic lipid overload, and *Apob* remains suppressed despite the metabolic need for VLDL export capacity. This suggests that impaired HNF1A function, due to insufficient protein stabilization by Hnf1aos1, may interfere with lipid-sensing regulation of *Lpl* and/or maintenance of *Apob* expression in response to hepatic lipid saturation. The opposing transcriptional changes amplify the triglyceride accumulation initiated by elevated uptake genes (*Cd36* and *Pparg*), creating a multifactorial steatotic phenotype in which excessive uptake is compounded by impaired export capacity.

This hepatic lipid trap mechanism is distinct from the primary uptake-driven defect mediated by the HNF1A-PPARG axis, representing a secondary metabolic consequence that exacerbates steatosis. Whether *Lpl* and *Apob* are direct HNF1A transcriptional targets or are indirectly regulated through impaired feedback signaling remains to be determined and requires ChIP-seq analysis and functional assessment of VLDL secretion and lipoprotein lipase activity.

### 3.7. Epigenetic Regulation—A Speculative Mechanism Based on mRNA Data

We observed elevated *Ezh2* expression specifically in Chow/Kd mice. However, the evidence argues against a primary epigenetic role: *Ezh2* mRNA normalizes under HFD conditions despite the more pronounced metabolic phenotype occurring in HFD/Kd mice. This temporal dissociation indicates that epigenetic dysregulation is unlikely to be the primary driving mechanism for HFD-induced steatosis. Additionally, we did not measure H3K27me3 patterns, confirm Hnf1aos1-EZH2 interactions, or quantify EZH2 protein levels, preventing definitive assessment of epigenetic involvement.

One highly speculative possibility, which is not supported by the current data, is that Hnf1aos1 might normally scaffold PRC2-mediated repression of metabolic genes such that its loss could reduce H3K27me3 deposition. The Ezh2 upregulation observed exclusively in Chow/Kd mice could conceivably represent an unsuccessful compensatory response. However, this interpretation is inconsistent with the observation that Ezh2 mRNA normalizes under HFD conditions precisely when the steatotic phenotype is most severe—directly arguing against a primary epigenetic mechanism driving hepatic lipid dysregulation. Without EZH2 protein quantification, H3K27me3 ChIP-seq, or Hnf1aos1–PRC2 co-immunoprecipitation data, this model cannot be substantiated and is presented only to acknowledge a possible alternative pathway [52,53]. Alternative explanations, such as secondary metabolic signaling responses, cannot be excluded.

To substantiate any epigenetic involvement, future studies would require (1) ChIP-seq to quantify H3K27me3 enrichment at metabolic gene loci; (2) co-immunoprecipitation or RNA immunoprecipitation to test Hnf1aos1–PRC2 physical interactions; and (3) EZH2 protein quantification to assess whether mRNA changes translate to functional alterations in PRC2 activity. None of these assays were performed in the present study. Given the poor correlation between *Ezh2* mRNA expression and steatosis severity, as well as the absence of any chromatin- or protein-level evidence, epigenetic mechanisms via PRC2 are unlikely to be the primary driver of HFD-induced steatosis and should not be interpreted as a mechanistic conclusion of this work.

### 3.8. Limitations

Limitations of the present study should be noted. First, and most importantly, HNF1A protein levels were not directly measured. The central mechanistic claim that Hnf1aos1 stabilizes the HNF1A protein to maintain repression of *Pparg* and *Cd36* during nutrient excess is based on transcriptional dissociation data and an analogy to the human HNF1A-AS1 system. Whether reduced HNF1A protein abundance is the proximate cause of the observed lipid phenotypes cannot be established without Western blot-, immunofluorescence-, or mass spectrometry-based quantification. These experiments are planned for future work. Second, the degree of hepatic Hnf1aos1 knockdown was partial (~30% under chow conditions and ~40% under HFD conditions), reflecting the mosaic nature of shRNA-mediated silencing via AAV delivery in vivo. Because knockdown was incomplete, the observed phenotypes likely underestimate the full metabolic consequence of Hnf1aos1 loss; a complete knockout model would provide stronger causal inference. Third, no chromatin-level assays (ChIP-seq and ATAC-seq) or protein–RNA interaction assays (RIP and eCLIP) were performed. Mechanistic claims regarding HNF1A protein stabilization, Ezh2/PRC2-mediated epigenetic regulation, and PPARG promoter occupancy are therefore inferential and require direct experimental support. Fourth, this study was conducted exclusively in male C57BL/6J mice, limiting generalizability across sexes and genetic backgrounds. Fifth, the sample size of *n* = 5 per group provided adequate power for the primary lipid endpoints but limits statistical confidence for secondary analyses, particularly those with *p*-values in the 0.06–0.08 range (*Cyp7a1*, TG:TC ratio).

### 3.9. Conclusions

This study identifies Hnf1aos1 as a previously unrecognized regulator of hepatic metabolic feedback control. Hnf1aos1 knockdown is associated with failure to suppress lipid uptake genes during nutrient excess, consistent with impaired HNF1A-mediated transcriptional control, producing a distinctive MASLD phenotype characterized by an elevated hepatic TG:TC ratio (6.1:1) and selective failure to suppress triglyceride uptake despite lipid saturation. These findings support a mechanistic model in which transcription factor mRNA abundance alone is insufficient for metabolic control. Functional stabilization through lncRNA cofactors appears to be required, although this principle will need to be confirmed with protein-level experiments.

This principle has translational significance. Human GWASs identify *HNF1A-AS1* variants as risk loci for dyslipidemia and MASLD, yet the functional basis for this genetic association remains unknown. Our mechanistic framework, in which Hnf1aos1 is proposed to stabilize the HNF1A protein to enable metabolic feedback control, provides a testable hypothesis for human disease mechanisms. Whether HNF1A-AS1 dysfunction contributes to MASLD progression in patients, as well as whether this regulatory axis represents a therapeutic target, requires functional validation in human hepatocytes and human genetic studies. Future work combining protein-level analysis, chromatin mapping, and functional assays of lipid transport in both murine and human systems will determine the clinical relevance of Hnf1aos1-mediated metabolic control.

## 4. Materials and Methods

### 4.1. AAV Vector Preparation and Administration

Recombinant adeno-associated virus serotype 8 (rAAV8) expressing short hairpin RNA (shRNA) targeting *Hnf1aos1* (rAAV8-*Hnf1aos1*; 5′-TACTGTAAAGCGTGTTTATTACTCGAGTAATAAACACGCTTTACAGTA-3′) or a scrambled control (rAAV8-Scramble; 5′-CCTAAGGTTAAGTCGCCCTCGCTCGAGCGAGGGCGACTTAACCTTAGG-3′) was purchased from VectorBuilder (Chicago, IL, USA). The viral titer was 1 × 10^12^ viral genome copies/mL (vg/mL), as determined by qPCR quantification by the manufacturer. The shRNA targeting Hnf1aos1 was designed to target exon 2 of the murine Hnf1aos1 transcript (NCBI RefSeq NR_027324.1), which is conserved across commonly used C57BL/6 strains. The scrambled control sequence was verified to have no predicted off-target homology to the murine transcriptome using NCBI BLAST+ (version 2.16.0 or later). Both constructs were driven by a U6 promoter within the AAV8 backbone and co-expressed with EGFP under the CMV promoter to permit in vivo verification of hepatic transduction. The AAV8 serotype provides efficient hepatic transduction with minimal off-target effects [54], and 5-week-old male C57BL/6J mice from Jackson Laboratory (Bar Harbor, ME, USA) received a total dose of 1 × 10^11^ vg (100 μL of 1 × 10^12^ vg/mL stock) via tail vein injection (*n* = 5 per group).

### 4.2. Animal Housing and Dietary Intervention

Mice were housed under standard conditions (12 h light/dark cycle, 22 ± 2 °C, 50–60% humidity, and *ad libitum* access to food and water). After a one-week acclimatization period, mice were randomized to either a standard chow diet (10% kcal from fat; Envigo Teklad 2018, Madison, WI, USA) or a high-fat diet (HFD; 60% kcal from fat; Bio-Serv S3282, Flemington, NJ, USA) for 12 weeks. Twelve weeks of a 60% kcal HFD is a widely used duration to induce MASLD-like steatosis and metabolic dysfunction in mice [55,56]. Body weight was monitored weekly. All animal procedures were approved by the University of Connecticut Institutional Animal Care and Use Committee (Protocol #A25-029) and conducted in accordance with the NIH Guide for the Care and Use of Laboratory Animals.

### 4.3. Serum Collection and Biochemistry

Blood was collected at the end of the study (week 12) via a cardiac puncture. Serum was separated by allowing the blood to clot at room temperature for 30 min, followed by centrifugation at 2000× *g* for 10 min at 4 °C, and stored at −80 °C. Serum triglycerides, cholesterol, HDL, and LDL were quantified by IDEXX BioAnalytics (North Grafton, MA, USA) using automated clinical chemistry analysis (n = 4 per group; one sample per group was excluded due to insufficient sample volume). The serum alanine aminotransferase (ALT) level was measured using a colorimetric assay kit (Cayman Chemical, Ann Arbor, MI, USA; #700260) according to the manufacturer’s instructions, read at 550 nm on a Synergy H1 microplate reader (BioTek, now Agilent Technologies, Santa Clara, CA, USA), and expressed as U/L based on a pyruvate standard curve (0–2 mM).

### 4.4. Tissue Collection and Organ Weighing

At study termination (12 weeks post-AAV administration), the mice were euthanized by CO_2_ inhalation followed by cervical dislocation. The liver was rapidly extracted, blotted, and weighed. The liver-to-body weight ratio was calculated and expressed as a percentage. Liver tissues were either snap-frozen in liquid nitrogen for biochemical analysis or fixed in 10% neutral-buffered formalin for histological analysis.

### 4.5. Hepatic Lipid Quantification

Liver triglyceride content was measured using the RayBio^®^ Triglyceride Colorimetric Assay Kit (RayBiotech, #MA-TG). Frozen liver tissue (~30 mg) was homogenized in ice-cold phosphate-buffered saline (PBS) containing protease inhibitors at a 1:10 (*w*/*v*) ratio and centrifuged at 14,000× *g* for 10 min at 4 °C. The supernatant was diluted with the assay’s Sample Buffer as required. Standards (0–200 mg/dL) and samples (10 µL) were added to 96-well plates in triplicate. After addition of the triglyceride reaction mix, the plates were incubated at room temperature for 30 min. The absorbance was measured at 510 nm. Triglyceride concentrations were calculated from the standard curves and expressed as mg/g of liver tissue.

Hepatic cholesterol was quantified using the RayBio^®^ Total Cholesterol Colorimetric Assay Kit (RayBiotech, #MA-TC). The liver tissue preparation step was identical to that used for the triglyceride assay. Standards (0–517.2 µM) and samples (10 µL) were added to 96-well plates in triplicate. The pre-warmed Enzyme Mix Solution (200 µL) was added, and the plates were incubated at 37 °C for 5 min. The absorbance was immediately measured at a wavelength of 500 nm. Cholesterol concentrations were calculated using standard curves, converted to mg/g using the manufacturer’s conversion factor, and expressed as mg/g of liver tissue.

### 4.6. Histological Analysis

Liver tissues were sectioned at 10 µm (H&E) or 8 µm (ORO) using a cryostat (Leica CM3050 S) and mounted on Superfrost Plus slides. Tissues were embedded in an optimal cutting temperature (OCT) compound and snap-frozen prior to cryosectioning. For H&E staining, sections were allowed to air-dry for 30 min prior to staining. For Oil Red O staining, frozen sections were brought to room temperature and fixed briefly before staining.

For H&E staining, sections were stained with Harris Hematoxylin for 10 s, rinsed, counterstained with 1% Eosin Y for 30 s, dehydrated with graded ethanol, cleared in xylene, and coverslipped with Permount. Histopathological evaluation was performed independently for each slide, with 10 high-power fields (HPFs) evaluated per slide. Sections were assessed for (1) hepatocellular lipid accumulation/degeneration with a microvesicular pattern, (2) hepatocellular lipid accumulation/degeneration with a macrovesicular pattern, (3) liver inflammation (periportal and interstitial), (4) hepatic fibrosis, and (5) hepatic necrosis. Each feature was scored on a 0–5 scale (0 = absent, 1 = minimal, 2 = mild, 3 = moderate, 4 = marked, and 5 = severe) per high-power field, and the final score per mouse for each feature represents the mean of the 10 HPFs evaluated per slide.

For ORO staining, sections were fixed in 10% neutral-buffered formalin for 10 min, incubated in 60% isopropanol, stained with a freshly filtered Oil Red O solution (0.5 g in 100 mL of isopropanol and diluted 6:4 with distilled water) for 10–15 min, rinsed with 60% isopropanol and distilled water, and counterstained with hematoxylin. The lipid droplet area and intensity were quantified using ImageJ software (version 1.8.0, National Institutes of Health, Bethesda, MD, USA) across 10 HPFs per slide, and the mean value was used for each animal.

Histopathological evaluation was performed by a board-certified veterinary pathologist (Department of Pathobiology, University of Connecticut) blinded to group assignments.

### 4.7. Reverse Transcription Quantitative PCR

Total RNA was isolated from frozen liver tissues using the TRIzol reagent (Thermo Fisher Scientific, Waltham, MA, USA) and a Precellys 24 homogenizer (Bertin Technologies, Montigny-le-Bretonneux, France). The RNA purity and concentration were determined using a NanoDrop spectrophotometer (Thermo Fisher Scientific, Waltham, MA, USA). Complementary DNA was synthesized from 1 µg of total RNA using an iScript cDNA Synthesis Kit (Bio-Rad Laboratories, Hercules, CA, USA). Quantitative real-time PCR was performed on a CFX96 Real-Time System (version 3.1; Bio-Rad Laboratories, Hercules, CA, USA) using the iTaq Universal SYBR Green Supermix (Bio-Rad Laboratories, Hercules, CA, USA) with gene-specific primers (Integrated DNA Technologies, Coralville, IA, USA; sequences are listed in Table 3). Gene expression was normalized using *Ppia* as an internal reference. Relative transcript abundance was calculated using the 2^−ΔΔCt^ method and expressed as fold change relative to the Chow/Scr group, which served as the common baseline for all four groups.

### 4.8. In Vivo Imaging System (IVIS)

AAV-mediated hepatic transgene expression was confirmed at the study’s endpoint (12 weeks) by in vivo fluorescence imaging. Anesthetized mice were imaged using an IVIS Spectrum system (PerkinElmer, Waltham, MA, USA) with excitation/emission filters appropriate for EGFP (excitation: 465 nm and emission: 520 nm). Hepatic fluorescence was quantified as total radiant efficiency ([p/s]/[μW/cm^2^]) in the liver region of interest using Living Image software (version 4.7.4; PerkinElmer, Waltham, MA, USA) and normalized to the background signal.

### 4.9. Statistical Analysis

All data are presented as the means ± standard deviations (SDs). Statistical analyses were performed using GraphPad Prism version 9.5.0 (GraphPad Software, San Diego, CA, USA). The specific statistical approach for each dataset is as follows.

Longitudinal body weight trajectories were analyzed using a linear mixed-effects model with restricted maximum likelihood (REML) estimation to account for repeated measurements within individual mice over the 12-week study period. The model included time (weeks 0–12) as a continuous within-subject factor, the experimental group (diet × genotype: Chow/Scr, Chow/Kd, HFD/Scr, HFD/Kd) as a between-subject factor, and their interaction (time × group) as fixed effects. Individual mouse identity was included as a random intercept to account for within-subject correlation. Model fit was assessed using the likelihood ratio test (chi-squared test). Significant main effects and interactions were determined by Fisher’s least significant difference (LSD) post hoc test for pairwise comparisons.

All terminal endpoint measurements were analyzed using ordinary one-way ANOVA to compare the four experimental groups (Chow/Scr, Chow/Kd, HFD/Scr, and HFD/Kd), followed by Fisher’s LSD post hoc test for multiple comparisons. The endpoint analyses included the (1) absolute liver weight and liver-to-body weight ratio; (2) hepatic lipid content (triglycerides and cholesterol); (3) serum biochemistry (alanine aminotransferase [ALT], cholesterol, LDL cholesterol, HDL cholesterol, and triglycerides); (4) quantitative histological assessment (Oil Red O-positive lipid area and intensity); (5) semi-quantitative histopathology scores (microvesicular steatosis, macrovesicular steatosis, inflammation, fibrosis, and necrosis); and (6) hepatic gene expression by quantitative RT-qPCR. The hepatic triglyceride-to-cholesterol ratio (TG:TC) was calculated for each mouse as individual TG (mg/g) divided by TC (mg/g), and group ratios were analyzed by one-way ANOVA followed by Fisher’s LSD. For gene expression data, fold-change values were calculated using the 2^−ΔΔCt^ method relative to Chow/Scr, which was used as the reference group, and then log_2_-transformed prior to one-way ANOVA.

Statistical significance was defined as * *p* < 0.05, ** *p* < 0.01, *** *p* < 0.001, and **** *p* < 0.0001. All statistical tests were two-tailed.

## Figures and Tables

**Table 1:** Semi-quantitative histopathological assessment of hepatic tissue.

Group	Microvesicular Steatosis	Macrovesicular Steatosis	Inflammation	Fibrosis	Necrosis	ORO
Chow/Scr	1.5	0	0.5	0	0	0.5
	1	0	0.5	0	0	0.15
	0.5	0	2	0	0	0.15
	0.5	0	2.5	0	0	0.5
	0.5	0	0.5	0	0	0.5
Chow/Kd	0.5	0	0.5	0	0	0.5
	0.5	0	1.5	0	0	0.15
	0.5	0	1.5	0	0	0.15
	1.5	0	2	0	0	0.5
	0.5	0	0	0	0	0.5
HFD/Scr	3.5	1	0.5	0	0	3.5
	2	0.5	0.5	0	0	2
	4	3	0.5	0	0	4
	1.5	2	0.5	0	0	1.5
	2.5	1.5	0.5	0	0	2.5
HFD/Kd	2.5	1.5	0.5	0	0	3
	4.5	3	0.5	0	0	4
	4.5	3	0.5	0	0	4.5
	3.5	3	0.5	0	0	4
	4.5	3	0.5	0	0	4

Scoring scale: 0 = absent, 1 = minimal, 2 = mild, 3 = moderate, 4 = marked, 5 = severe with mean ± SD of the 0–5 scores per group. Values shown are per-mouse mean scores across 10 high-power fields, with *n* = 5 per group. As these are averages, half-step values may occur.

**Table 2:** TG:TC values corresponding to Figure 2H.

Group	Hepatic TG (mg/g)	Hepatic Cholesterol (mg/g)	TG:TC Ratio
Chow/Scr	15.05 ± 4.5	3.51 ± 1.1	4.3:1
Chow/Kd	16.45 ± 11.0	6.10 ± 2.9	2.7:1
HFD/Scr	26.34 ± 11.9	6.67 ± 0.6	4.0:1
HFD/Kd	51.72 ± 19.8	8.47 ± 2.5	6.1:1

**Table 3:** Primers used for RT-qPCR.

Gene	Forward Primer (5′→3′)	Reverse Primer (5′→3′)
*Hnf1aos1*	GACTGTCCCCAAACTCCTCA	TTCCCTGAGATTATCGCCCG
*Hnf1a*	AGAGACCTTGGTGGAGGAGTGT	GGCAAACCAGTTGTAGACACGC
*Cyp7a1*	AACAACCTGCCAGTACTAGATAGC	GTGTAGAGTGAAGTCCTCCTTAGC
*Cd36*	GGACATTGAGATTCTTTTCCTCTG	GCAAAGGCATTGGCTGGAAGAAC
*Pparg*	GTACTGTCGGTTTCAGAAGTGCC	ATCTCCGCCAACAGCTTCTCCT
*Srebf1*	CGACTACATCCGCTTCTTGCAG	CCTCCATAGACACATCTGTGCC
*Fasn*	CACAGTGCTCAAAGGACATGCC	CACCAGGTGTAGTGCCTTCCTC
*Apob*	GCATGAGTATGCCAATGGTCTCC	CTGGTTGCCATCTGAAGCCATG
*Lpl*	GCGTAGCAGGAAGTCTGACCAA	AGCGTCATCAGGAGAAAGGCG
*Abca1*	GGAGCCTTTGTGGAACTCTTCC	CGCTCTCTTCAGCCACTTTGAG
*Vldlr*	ACGGCAGCGATGAGGTCAACTG	CAGAGCCATCAACACAGTCTCG
*Ezh2*	CATACGCTCTTCTGTCGACGATG	ACACTGTGGTCCACAAGGCTTG
*Srebf2*	AGAAAGAGCGGTGGAGTCCTTG	GAACTGCTGGAGAATGGTGAGG
*Ppia*	CATACAGGTCCTGGCATCTTGTC	AGACCACATGCTTGCCATCCAG
*Fabp4*	TGAAATCACCGCAGACGACAGG	GCTTGTCACCATCTCGTTTTCTC
*Ldlr*	TTGGGGAACACCCGCCAAGA	TCCGATTGCCCCCATTGACA
*Lxra*	ATCGCCTTGCTGAAGACCTCTG	CTGCTTTGGCAAAGTCTTCCCG
*Acaca*	GTTCTGTTGGACAACGCCTTCAC	GGAGTCACAGAAGCAGCCCATT
*Ppara*	ACCACTACGGAGTTCACGCATG	GAATCTTGCAGCTCCGATCACAC

## Data Availability

All raw data and processed data are stored in the OneDrive of Zhong laboratory at the University of Connecticut. The data are available to the public. The individual repository accession numbers are listed below. When published, all raw data and processed data will also be deposited to a NIGMS-dedicated repository that followed the NIGMS Data Management and Sharing Plan policy. 1. Quantitative RT-qPCR data (fold-change values for all genes tested and corresponding Ct values) were uploaded to Mendeley Data. [DOI: 10.17632/hp5wwtjsnz.1]. 2. Hepatic lipid quantification data (triglyceride and cholesterol measurements in mg/g tissue, raw absorbance values, and standard curves) were uploaded to Mendeley Data. [DOI: 10.17632/hdx6wnxfw7.1]. 3. Serum biochemistry data (ALT, triglyceride, cholesterol, HDL cholesterol, and LDL cholesterol measurements) were uploaded to Mendeley Data. [DOI: 10.17632/32v77h2sbv.1]. 4. Histopathology image files and quantitative analysis (H&E and Oil Red O staining images, semi-quantitative histopathology scores for all five features, and lipid area/intensity quantification) were uploaded to Mendeley Data. [DOI: 10.17632/j5yrpkgcpb.1]. 5. Body weight data (weekly measurements throughout the 12-week study period) were uploaded to Mendeley Data. [DOI: 10.17632/jdhn74k7gt.1].

## Supplementary Materials

The following supporting information can be downloaded at: https://www.mdpi.com/article/10.3390/ncrna12030015/s1, Figure S1: Extended transcriptional profiling of hepatic lipid regulatory genes in chow- and high-fat diet-fed Hnf1aos1 knockdown and control mice.

## Abbreviations

AAV	adeno-associated virus
ABCA1	ATP-binding cassette transporter A1
ACC	acetyl-CoA carboxylase
Acaca	acetyl-CoA carboxylase alpha
Akt2	protein kinase B beta
ALT	alanine aminotransferase
AMPK	adenosine monophosphate-activated protein kinase
Apob	apolipoprotein B
AUC	area under the curve
CO_2_	Carbon Dioxide
Cd36	cluster of differentiation 36
ChIP	chromatin immunoprecipitation
Cyp7a1	cytochrome P450 family 7 subfamily A member 1
Ezh2	Enhancer of Zeste Homolog 2
Fabp4	fatty acid binding protein 4
Fasn	fatty acid synthase
GWAS	genome-wide association study
H&E	hematoxylin and eosin
H3K27me3	histone H3 lysine 27 trimethylation
HFD	high-fat diet
HDL	high-density lipoprotein
HNF1A	hepatocyte nuclear factor 1 alpha
HNF1A-AS1	HNF1A antisense RNA 1 (human lncRNA)
Hnf1aos1	HNF1A opposite strand 1 (mouse ortholog of HNF1A-AS1)
HPF	High-power field
IDEXX	IDEXX Laboratories
ImageJ	Image analysis software
IVIS	in vivo imaging system
Lcat	lecithin-cholesterol acyltransferase
Ldlr	low-density lipoprotein receptor
LDL	low-density lipoprotein
lncRNA	long noncoding RNA
Lpl	lipoprotein lipase
Lxra	liver X receptor alpha
LXR	liver X receptor
MASLD	metabolic dysfunction-associated steatotic liver disease
MODY3	maturity-onset diabetes of the young type 3
NAFLD	nonalcoholic fatty liver disease
OCT	Optimal cutting temperature compound
ORO	Oil Red O
PBS	phosphate-buffered saline
Ppara	peroxisome proliferator-activated receptor alpha
Pparg	peroxisome proliferator-activated receptor gamma
Ppia	peptidylprolyl isomerase A
PRC2	Polycomb Repressive Complex 2
qPCR	quantitative polymerase chain reaction
rAAV8	recombinant adeno-associated virus serotype 8
REML	Restricted maximum likelihood
RT-qPCR	reverse transcription quantitative polymerase chain reaction
Scap	SREBP cleavage-activating protein
SD	standard deviation
Srebf1	sterol regulatory element-binding transcription factor 1
Srebf2	sterol regulatory element-binding transcription factor 2
SREBP	sterol regulatory element-binding protein
TG:TC	triglyceride-to-cholesterol
TRIM25	tripartite motif containing 25
Vldlr	very-low-density lipoprotein receptor
