# Supplementary material for: Hnf1aos1 as a Metabolic Coordinator of Hepatic Lipid Homeostasis and Feedback Control
Source: Noncoding RNA. 2026 Apr 30;12(3):15. doi: 10.3390/ncrna12030015 (PMC13214671; doi:10.3390/ncrna12030015)
Supplement: Supplementary file 1 [file ncrna-12-00015-s001.zip › ncrna-4241349-supplementary.pdf]

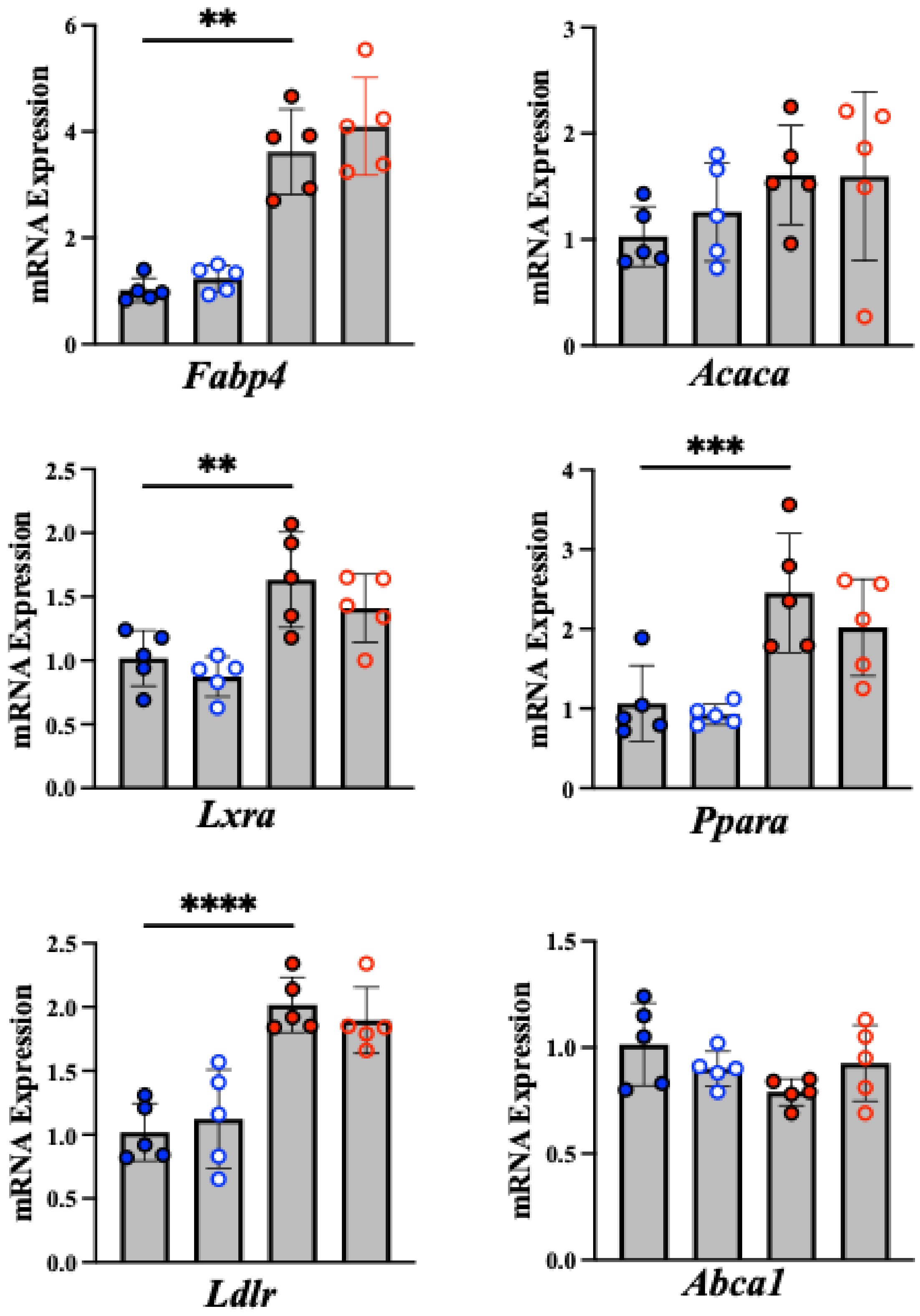

Supplementary Figure S1. Extended transcriptional profiling of hepatic lipid regulatory genes in Chow- and High-fat diet (HFD)–fed Hnf1aos1 knockdown and control mice. Relative mRNA expression of *Fabp4*, *Acaca*, *Lxra*, *Ppara*, *Ldlr*, and *Abca1* was quantified by RT-qPCR in liver tissues after 12 weeks of dietary intervention. Data are presented as mean ± SD (n = 5 per group; one-way ANOVA followed by Fisher's LSD; ***p*<0.01, ****p*<0.001, *****p*<0.0001.
